# Dynamics of photosynthetic induction and relaxation within the canopy of rice and two wild relatives

**DOI:** 10.1002/fes3.286

**Published:** 2021-05-05

**Authors:** Liana G. Acevedo‐Siaca, Jacqueline Dionora, Rebecca Laza, William Paul Quick, Stephen P. Long

**Affiliations:** ^1^ Department of Crop Sciences University of Illinois at Urbana‐Champaign Urbana IL USA; ^2^ Carl R. Woese Institute for Genomic Biology University of Illinois at Urbana‐Champaign Urbana IL USA; ^3^ Global Wheat Program, International Maize and Wheat Improvement Center (CIMMYT) Mexico DF Mexico; ^4^ C_4_ Rice Center International Rice Research Institute Los Baños Philippines; ^5^ Department of Animal and Plant Sciences University of Sheffield Sheffield UK; ^6^ Department of Plant Biology University of Illinois at Urbana‐Champaign Urbana IL USA; ^7^ Lancaster Environment Centre Lancaster University Lancaster UK

**Keywords:** crop canopy dynamics, non‐photochemical quenching, non‐steady‐state photosynthesis, photosynthetic induction, rice

## Abstract

Wild rice species are a source of genetic material for improving cultivated rice (*Oryza sativa*) and a means to understand its evolutionary history. Renewed interest in non‐steady‐state photosynthesis in crops has taken place due its potential in improving sustainable productivity. Variation was characterized for photosynthetic induction and relaxation at two leaf canopy levels in three rice species. The wild rice accessions had 16%–40% higher rates of leaf CO_2_ uptake (*A*) during photosynthetic induction relative to the *O*. *sativa* accession. However, *O*. *sativa* had an overall higher photosynthetic capacity when compared to accessions of its wild progenitors. Additionally, *O*. *sativa* had a faster stomatal closing response, resulting in higher intrinsic water‐use efficiency during high‐to‐low light transitions. Leaf position in the canopy had a significant effect on non‐steady‐state photosynthesis, but not steady‐state photosynthesis. The results show potential to utilize wild material to refine plant models and improve non‐steady‐state photosynthesis in cultivated rice for increased productivity.

## Graphical abstract

We characterized non‐steady state photosynthesis in an elite rice cultivar and single accessions of its two closest wild relatives and ancestors. The two wild accessions can adjust more rapidly and assimilate more CO_2_ during transitions from shade to full sunlight. This suggests considerable breeding potential in using this and broader biodiversity in improving rice productivity.

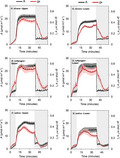

## 1 INTRODUCTION

Cultivated rice (*Oryza sativa*) plays a central role in ensuring global food security, especially throughout Asia where millions of people are dependent upon it for most of their daily caloric intake. Globally, it is the single most important direct source of human calories (Mohanty, 2013). Cultivated rice is believed to have been domesticated as early as 6000 BCE from wild progenitors *O*. *rufipogon* and *O*. *nivara*, whose distribution stretches throughout the Asian continent and into northern Australia (Choi & Purugganan, 2018; Garris et al., 2005; Molina et al., 2011; Silva et al., 2015; Sweeney & McCouch, 2007). Both wild rice species are semi‐aquatic and grow in varied ecosystems, ranging from drier land to permanent semi‐submerged conditions, such as pools or ditches (Mohapatra et al., 2011). *O*. *rufipogon* and *O*. *nivara* tend to be weedy and invasive, establish many tillers, and grow taller in height than cultivated rice (Mohapatra et al., 2011). While these traits may be beneficial in the wild where they will serve to shade out competitors, they do not lend themselves to an agricultural setting where inter‐plant competition reduces yield (Zhu et al., 2010). However, over thousands of years of selection (Gross & Zhao, 2014; Zheng et al., 2016) cultivated rice began to more closely resemble the varieties that much of humankind depends on today.

During the 20th century, physiological traits beneficial to rice productivity were selected to create an “idealized plant type” or “ideotype.” The rice ideotype is shorter, has erect leaves with steep leaf angles, and fewer tillers (Dingkhun et al., 1991). Steeper leaf angles allow greater infiltration and more even distribution of light through the canopy, reducing photoinhibition and leaf temperatures (Burgess et al., 2015; Dingkuhn et al., 1991; Falster & Westoby, 2003; Muchie et al., 1999; Werner et al., 2001). Fewer tillers reduce the occurrence of mutual shading of leaves within the canopy. Excessive tillering decreases net canopy photosynthesis and diverts resources from other organs, including from the panicle (Dingkhun et al., 1991). The combination of these traits leads to greater crop photosynthetic efficiency and yield (Burgess et al., 2017). Some of the most productive rice accessions, such as IR64 and IR72, were deliberately bred to improve light distribution within the canopy and can yield 8.7–9.5 tons per hectare in the absence of disease and given adequate water and nutrients (Mackill & Khush, 2018).

Despite these improvements, light throughout a plant canopy is not distributed equally and can vary greatly from bottom to top (Meacham et al., 2017; Zhu et al., 2000, 2004). Additionally, canopy light distribution can be affected by changes in cloud cover, wind, and self‐shading between leaves. As a result, canopy light environments are constantly changing, from sun to shade and shade to sun in seconds (Pearcy, 1990; Pearcy et al., 1994; Zhu et al., 2000, 2004; Slattery et al., 2018; Wang, Shi et al., 2020). Many understory plants and leaves in the lower canopy layers are dependent upon diffuse light or sunflecks, brief increases in solar radiation that can last seconds to minutes, to drive photochemistry (Chazdon & Pearcy, 1990; Pearcy, 1990; Pearcy & Chazdon, 1994; Zhu et al., 2000, 2004). Since high photon flux densities are not always available in the lower canopy, leaves must be able to respond quickly to fluctuating light to effectively drive photosynthesis. Photosynthetic induction is the process by which leaves begin to increase the assimilation of CO_2_ once they transition from low light (shade) to high light (sun). Photosynthetic induction is characterized by a lag in efficiency due to the regeneration of Ribulose 1, 5‐bisphosphate (RuBP), the buildup of carbon metabolite intermediates, activation of Ribulose 1, 5‐bisphosphate carboxylase/oxygenase (Rubisco), and stomatal opening as photosynthesis moves toward a steady‐state (Mott & Woodrow, 2000; Pearcy et al., 1994). Because leaf CO_2_ uptake rates are lower throughout induction than at steady state, this represents forgone assimilation. The amount forgone due to these lower rates during induction in a wheat canopy over the course of a season was calculated at 21% (Taylor & Long, 2017). Further, in cultivated rice, intrinsic water‐use efficiency (iWUE) is lower during induction, so causing an increase in water demand per CO_2_ assimilated (Acevedo‐Siaca et al., 2020a). However, when considering transient photosynthetic responses, induction is only one half of the equation.

When a leaf is in full sunlight, it is absorbing more light than it can utilize to drive photosynthesis. To avoid oxidative photodamage, the excess absorbed light energy is dissipated through a light‐induced process termed non‐photochemical quenching (NPQ) in which excess light energy from the leaf is dissipated as heat (Bradbury & Baker, 1983). However, when a leaf suddenly goes into the shade, NPQ continues even though all available light energy could now be used in photosynthesis. Over the course of minutes, leaf CO_2_ uptake rate increases in the shade to a new steady state as NPQ relaxes. Recently, much attention has been given to improving photoprotection and accelerating NPQ relaxation during sun to shade transitions (Kromdijk et al., 2016; Murchie et al., 2015; Murchie & Niyogi et al., 2011). Forgone carbon assimilation by crop canopies due to this slow relaxation is estimated to cost between 10% and 30% of daily total assimilation (Zhu et al., 2000, 2004). Indeed, biomass production was increased by 14%–21% in field‐grown tobacco that had been bioengineered to speed relaxation of NPQ (Kromdijk et al., 2016). Additionally, several studies have identified QTLs linked to NPQ performance that could be used to improve photosynthetic efficiency and crop productivity (Rungrat et al., 2019; Wang, Zhao, et al., 2020). Unlike photosynthetic induction, which is characterized by a lag in photosynthetic rates, the decrease in leaf CO_2_ uptake rate is almost instantaneous on transfer from sun to shade. However, stomatal conductance declines to a new steady‐state over the course of several minutes. As a result, *iWUE* is considerably lower for several minutes than at steady state during sun to shade transitions (McAusland et al., 2016; Qu et al., 2016). Improvements to both photosynthetic induction and relaxation would, therefore, result in both greater productivity and higher water‐use efficiency.

Despite photosynthesis in crops taking place in a dynamic environment, most measurements and understanding of the process in crops are within the context of controlled, steady‐state measurements. Yet, steady‐state lighting is seldom achieved in leaves within a field crop canopy. Additionally, previous models that utilize steady‐state data potentially overestimate CO_2_ assimilation and do not account for the loss of productivity due to the lags in efficiency during induction and relaxation (Pearcy, 1990; Taylor & Long, 2017; Wang, Burgess, et al., 2020) and will underestimate water loss (McAusland et al., 2016; Qu et al., 2016; McAusland et al., 2020). However, increased focus has recently been dedicated to characterizing, understanding, and modeling photosynthesis in non‐steady‐state conditions, to more accurately reflect conditions in the field (Kaiser et al., 2015; Kaiser et al., 2017; McAusland et al., 2016; Qu et al., 2016; Soleh et al., 2016, 2017; Kaiser et al., 2018; Deans et al., 2019; Deans et al., 2019; De Souza et al., 2020; Wang, Burgess, et al., 2020; Acevedo‐Siaca, Coe, Quick, et al., 2020; Acevedo‐Siaca, Coe, Wang, et al., 2020; McAusland et al., 2020). As a result, new targets for improving photosynthetic efficiency have been identified that could improve performance in non‐steady‐state conditions, such as increasing the speed of induction, reducing forgone assimilation during induction, reducing water loss, improving stomatal kinetics, or relaxing NPQ more quickly (Woodrow & Mott, 1989; Lawson & Blatt, 2014; McAusland et al., 2016; Kromdijk et al., 2016; Glowacka et al., 2018; Qu et al., 2016; De Souza et al., 2020; Acevedo‐Siaca, Coe, Quick, et al., 2020; Acevedo‐Siaca, Coe, Wang, et al., 2020; McAusland et al., 2020; Qu et al., 2020).

Wild rice species act as a source of germplasm for the improvement of cultivated rice. Indeed, several studies have aimed to characterize natural variation within the genus *Oryza* in the search of traits that might confer resistance to abiotic and biotic threats in cultivated rice (Sanchez et al., 2013; Song et al., 2005; Wang, Burgess, et al., 2020). Additionally, several studies have examined photosynthetic performance in wild rice species and in some cases have found higher rates for leaf CO_2_ uptake in wild rice relative to cultivated (Giuliani et al., 2013; Zhao et al., 2008). Furthermore, the potential for improving rice photosynthetic performance from wild germplasm has already been highlighted (Haritha et al., 2017). Although natural variation for photosynthesis has previously been studied in wild rice species, these have been within the context of steady‐state conditions. Prior work within cultivated rice showed almost threefold greater between accession variation in photosynthetic traits during induction compared to steady state (Qu et al., 2016; Acevedo‐Siaca, Coe, Quick, et al., 2020). This suggests that greater improvements in photosynthetic capacity and efficiency might be achieved with a focus on non‐steady‐state traits. This study examines these traits to (i) understand variation in both shade‐sun and sun‐shade transitions between accessions of *O*. *sativa*, *O*. *rufipogon*, and *O*. *nivara*; (ii) determine the effect of canopy level on this variation, and (iii) determine the factors affecting forgone assimilation during sun‐shade and shade‐sun transitions.

## 2 MATERIALS AND METHODS

### 2.1 Selected germplasm and growing conditions

Accessions from three rice species were selected: cultivated Asian rice (*O*. *sativa*) represented by IR64 (IRGC# 117268) and its wild progenitors (*O*. *rufipogon* and *O*.* nivara*, IRGC#s 126954 and 136116, respectively). The wild rice species were chosen due to their similarity in plant architecture relative to IR64, making comparisons between levels of the canopy more transferable. Rice accessions were provided by the International Rice Research Institute's (IRRI) Genetic Resources Center (GRC). Rice seed dormancy was broken by placing seeds in an oven at 50° Celsius for one week. Seeds were then germinated in Petri dishes prior to being transplanted to pots. Rice plants were grown at IRRI, Los Baños, in screenhouses, that is, a greenhouse that has meshed walls that allow circulation of air between the enclosure and the outside environment, without additional temperature control or lighting. Pots were maintained flooded to simulate paddy conditions. Measurements took place during the Philippines rainy season from July to September 2019. Average day/night temperatures during this period at Los Baños are 31°C/23°C, with a daytime relative humidity of ≥70%.

### 2.2 Gas exchange measurements and chlorophyll fluorescence

Steady‐ and non‐steady‐state photosynthesis were measured on six plants of each accession (*n* = 6). Photosynthesis was measured at two canopy levels (low and high) on vegetative leaves. Leaves at the “high” canopy level were the youngest fully expanded on a primary tiller that was not shaded by surrounding leaves in the canopy. The leaf at the “low” canopy level was also a youngest fully expanded leaf from a tiller but lower in the canopy and shaded by other leaves. Gas exchange measurements were made over the course of a week from 08:00 to 13:00, to avoid confounding photosynthesis with any marked circadian effects. Additionally, plants were selected at random to avoid confounding species with time. Leaf CO_2_ uptake (*A*), stomatal conductance (*g*_s_), intercellular CO_2_ concentration ($C_{i}$), and intrinsic water‐use efficiency (*iWUE*) were calculated following the equations of von Caemmerer & Farquhar (1981) A full list of traits measured and their abbreviations are provided in Table 1.

**TABLE 1:** A summary of all traits measured and mentioned in the text. Units and light conditions are included

Light Condition	Trait	Description	Unit
Steady‐state	*A_sat_*	Leaf net CO_2_ uptake in saturating light	µmol m^−2^ s^−1^
	*g_s_*	Stomatal conductance	mol m^−2^ s^−1^
	*C_i_*	Intercellular CO_2_ concentration	µmol mol^−1^
	*iWUE*	Intrinsic water‐use efficiency (Iwue = A/*g_s_*)	µmol CO_2_ mol H_2_O^−1^
	*A_Max_*	CO_2_ uptake in saturating [CO_2_] and light	µmol m^−2^ s^−1^
	*V_c,_* *_max_*	Maximum rate of carboxylation	µmol m^−2^ s^−1^
	*J_max_*	Maximum rate of electron transport	µmol m^−2^ s^−1^
	*TPU*	Triosephosphate utilization	
	*CE*	Carboxylation efficiency	mol m^–2 ^s^–1^
	*Γ*	Compensation point	µmol m^−2^ s^−1^
	*Φ*	Quantum yield	unitless (0–1)
	*L*	Stomatal limitation	µmol mol^−1^
Non‐steady‐state	$\bar{A}$	Average *A* during induction	µmol m^−2^ s^−1^
	$\overset{‐}{g_{s}}$	Average *g_s_* during induction	mol m^−2^ s^−1^
	$\overset{‐}{C_{i}}$	Average *C_i_* during induction	µmol mol^−1^
	$\bar{\mathrm{iWUE}}$	Average iWUE during induction	µmol CO_2_ mol H_2_O^−1^
	*A_s_*	CO_2_ uptake after induction once steady‐state is reached	µmol m^−2^ s^−1^
	*IT_50_*	Time to 50% induction	Seconds
	*IT_90_*	Time to 90% induction	Seconds
	$g_{s\;50\;i}$	Time to 50% stomatal conductance during induction	Seconds
	$g_{s\;90\;i}$	Time to 90% stomatal conductance during induction	Seconds
	$g_{s\;50\;r}$	Time to 50% stomatal conductance during high‐light to low‐light transition	Seconds
	$g_{s\;90\;r}$	Time to 90% stomatal conductance during high‐light to low‐light transition	Seconds
Chlorophyll Fluorescence	*NPQ*	Non‐photochemical quenching	unitless
Leaf Traits	*LA*	Fresh leaf area	mm^−2^
	*DW*	Dry leaf weight	g^−1^
	*SLA*	Specific leaf area	mm^−2^ g^−1^

#### 2.2.1 Steady‐state measurements

Photosynthesis and chlorophyll fluorescence were measured using an infra‐red gas analyzer (IRGA) inside of the screenhouse on the leaves described previously (LI6400‐XT, LI‐COR Biosciences, Lincoln, NE, USA). Light was provided through an integrated LED light source and modulated fluorometer (2 cm^2^, LI‐6400‐40, LI‐COR Environmental, Lincoln, NE, USA). Within the cuvette, the air temperature was 28°C, the flow rate was 400 µmol s^−1^_,_ [CO_2_] was maintained at 400 µmol mol^−1^, and water vapor pressure deficit (VPD) at 1.4–1.8 kPa. For steady‐state measurements, leaves were allowed to reach constant *A* and *g_s_* at 1,500 µmol m^−2^ s^−1^ PPFD. The maximum quantum yield of photosystem II (F_v_/F_m_) was also measured once the leaves reached a steady‐state using the multiphase flash procedure (Loriaux et al., 2013).

Additionally, the response of *A* to intercellular CO_2_ concentration (*C_i_*) was measured on six plants per accession at both canopy levels (*n* = 6; 12 measurements total/accession). Photosynthesis was measured at saturating light (1,500 µmol m^−2^ s^−1^) and at the following [CO_2_]: 400, 40, 70, 100, 200, 300, 400, 800, 1,000, 1,500, and 1,800 µmol mol^−1^ in this sequence but waiting for steady‐state *A* to be achieved at each step. These measurements were then used to construct *A*/*C_i_* curves and solve for the maximum rate of carboxylation (*V_c_*_,max_), the maximum rate of electron transport (*J*_max_), triosephosphate utilization (*TPU*), carboxylation efficiency (*CE*), and CO_2_ compensation point (*Γ*), and maximum rate of CO_2_ uptake in saturating light and [CO_2_] (*A*_max_) following the methods of Bernacchi et al. (2003) and Long and Bernacchi (2003).

Stomatal limitation (*L*) was calculated from the *A*/*C_i_* curves following the equation of Long and Bernacchi (2003):

(1) $$L=\left(A^{\prime \prime }‐A^{\prime }\right)/A^{\prime \prime }$$

Where *A*′ is the operating point at an atmospheric [CO_2_] (*C_a_*) of 400 µmol mol^−1^ and *A*″ is the hypothetical *A* if *C_i_* = *Ca = 400* µmol mol^−1^; the case in the absence of stomatal limitation.

The response of CO_2_ uptake (*A*) to photosynthetic photon flux density (PPFD) was measured on six plants per accession at both canopy levels (*n* = 6; 12 measurements total/accession) (Figure S1). Photosynthesis was measured at ambient [CO_2_] (400 µmol mol^−1^) and at the following light intensities: 0, 20, 50, 100, 200, 400, 600, 1,000, 1,500, and 2,000 µmol m^−2^ s^−1^.

#### 2.2.2 Non‐steady‐state measurements: photosynthetic induction and relaxation

Leaves were dark‐adapted for at least 30 minutes prior to beginning gas exchange measurements and to obtain a minimum value of chlorophyll fluorescence (F_o_) and maximal possible value of chlorophyll fluorescence (F_m_). For induction, leaves were allowed to reach steady state in low light (50 µmol m^−2^ s^−1^ PPFD) for nine minutes followed by 30 minutes of high light (1,500 µmol m^−2^ s^−1^ PPFD). The dynamics of photosynthesis on a sun‐shade transition were studied by reducing the light intensity back to low light (50 µmol m‐2 s‐1 PPFD) for 21 minutes after the 30 minutes of high light. Gas exchange was logged every ten seconds, while modulated chlorophyll fluorescence parameters needed to estimate NPQ were taken every three minutes. This lower frequency of measurement was a compromise between determining NPQ versus avoiding significant re‐activation of NPQ by applying the multi‐flash procedure. Cuvette conditions for temperature, flow rate, [CO_2_], and VPD were as described above for measurements of steady‐state photosynthesis.

Photosynthetic traits in non‐steady‐state conditions were averaged over the first 20 minutes of induction such as average CO_2_ uptake ($\bar{A}$), average stomatal conductance (${\bar{g}}_{s}$), average intercellular CO_2_ concentration (${\bar{C}}_{i}$), and average intrinsic water‐use efficiency ($\bar{\mathrm{iWUE}}$). The speed of induction was calculated by measuring the time in seconds to 50% and 90% induction relative to the steady state for CO_2_ uptake (*IT_50_* and *IT_90_*, respectively). The initial slope during the first 5 minutes of induction, where the greatest change in CO_2_ uptake is seen, was also calculated to make comparisons between the induction responses of different accessions and canopy levels. The time to 50% and 90% steady‐state stomatal conductance was also measured during induction (*g_s_* *_50_* *_i_* and *g_s_* *_90_* *_i_*) to understand the rate of stomatal opening. Stomatal closure was examined by calculating the time to 50% and 90% steady‐state stomatal conductance after a transition from high light to low light (*g_s_* *_50_* *_r_* and *g_s_* *_90_* *_r_*, respectively).

### 2.3 Leaf area, weight, and specific leaf area

Upon completion of measurements of gas exchange and chlorophyll fluorescence, the measured leaves were immediately harvested to calculate leaf area (LA) and then dried to constant weight at 80°C to determine the dry weight (DW), and then calculate specific leaf area (SLA) for a single leaf. Leaf area was measured using an LI‐3100C Area Meter (LI‐COR Environmental).

### 2.4 Calculations

#### 2.4.1 Correcting photosynthesis for stomatal limitation and calculating forgone assimilation

*A* was corrected to remove stomatal limitation (A^*^) following the methods of Soleh et al., (2016) and Acevedo‐Siaca, Coe, Wang, et al., (2020). Where the intercellular CO_2_ concentration was determined from the values once a steady‐state was reached:

(2) $$A^{*}=A\times \frac{300}{C_{i}}$$

Non‐photochemical quenching (NPQ) was calculated following the equation described in Murchie and Lawson (2013):

(3) $$\text{NPQ}=\left(F_{m}‐F_{m}^{\prime }\right)/F_{m}^{\prime }$$

Where *F_m_* is the maximal fluorescence yield in a dark‐adapted, non‐stressed leaf, and *F′_m_* is the maximal fluorescence yield in a light‐adapted state (Murchie & Lawson, 2013). This equation estimates the rate constant for heat loss from PSII (Murchie & Lawson, 2013).

The quantum efficiency of PSII electron transport was also calculated following the equation of Murchie and Lawson (2013):

(4) $$\Phi_{\text{PSII}}=\left(F_{m}^{\prime }‐F_{s}^{\prime }\right)/F_{m}^{\prime }$$

Where *F′_s_* is the steady‐state level of fluorescence in the light (Murchie & Lawson, 2013). $\Phi_{\mathrm{PSII}}$ is the quantum yield of photosystem II (PSII), that is, the number of electrons entering whole chain electron transport at PSII per absorbed photon (Maxwell & Johnson, 2000).

### 2.5 *Statistical* *analyses*

The normality of data was evaluated utilizing the “qqp” QQ plot function with confidence intervals (R; “car” and “MASS”). Normality was met for all data and therefore parametric, analysis of variance (ANOVA) was performed (R; “lme4” and “lmerTest”). Where significance was obtained, Tukey's honest significant discrimination (HSD) analysis (R; “agricolae” “lawstat”) was applied. All statistical analyses were performed in R (version 3.3.2) (R Core Team, 2020) using the following model:

$$Y_{\text{ijk}}=\mu +S_{i}+C_{j}+{\text{SC}}_{\text{ij}}+\varepsilon_{\text{ijk}}$$

Where,

*Y*_ijk_ = the response of the trait of interest

µ = the grand mean

*S*_i_ = the fixed effect of the ith species

*C*_j_ = the random effect of jth canopy level

SC_ij_ = the random effect of the interaction of the ith species and jth canopy level

*ε*_ijk_ = the random effect of the error (NID = 0, σ)

## 3 RESULTS

### 3.1 Characterization of photosynthetic induction traits in three *Oryza species* accessions

**FIGURE 1:**
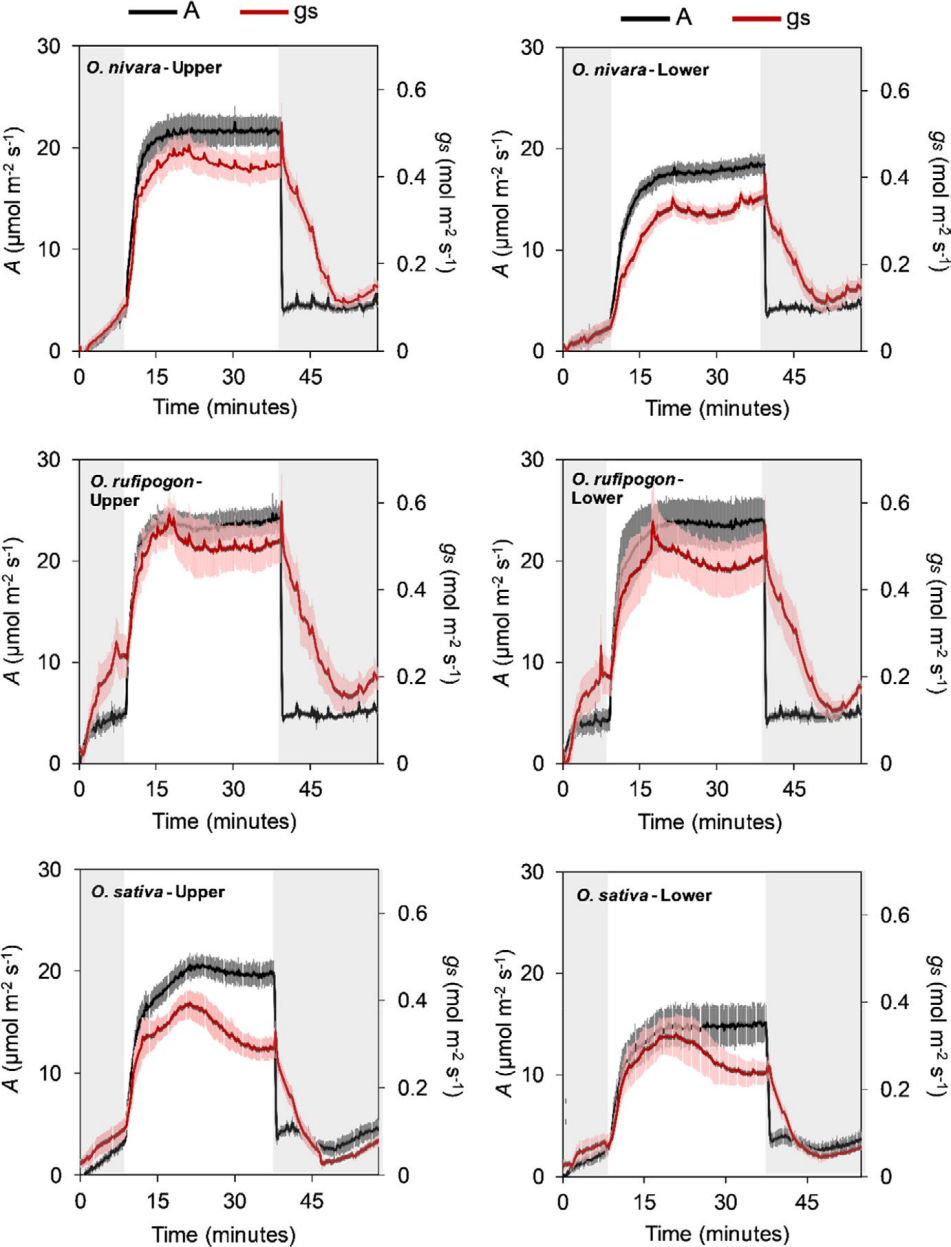
CO_2_ uptake (A) and stomatal conductance (*g_s_*) over time showing an increase during photosynthetic induction by high light (1,500 µmol m^−2^ s^−1^) followed by a decrease in response to low light (50 µmol m^−2^ s^−1^). Measurements were taken in three *Oryza* species and at two canopy levels in each plant (upper and lower). The measurement was taken at an ambient [CO_2_] of 400 µmol mol^−1^. Each point is the mean (±SE) of six plants (*n* = 6).

**FIGURE 2:**
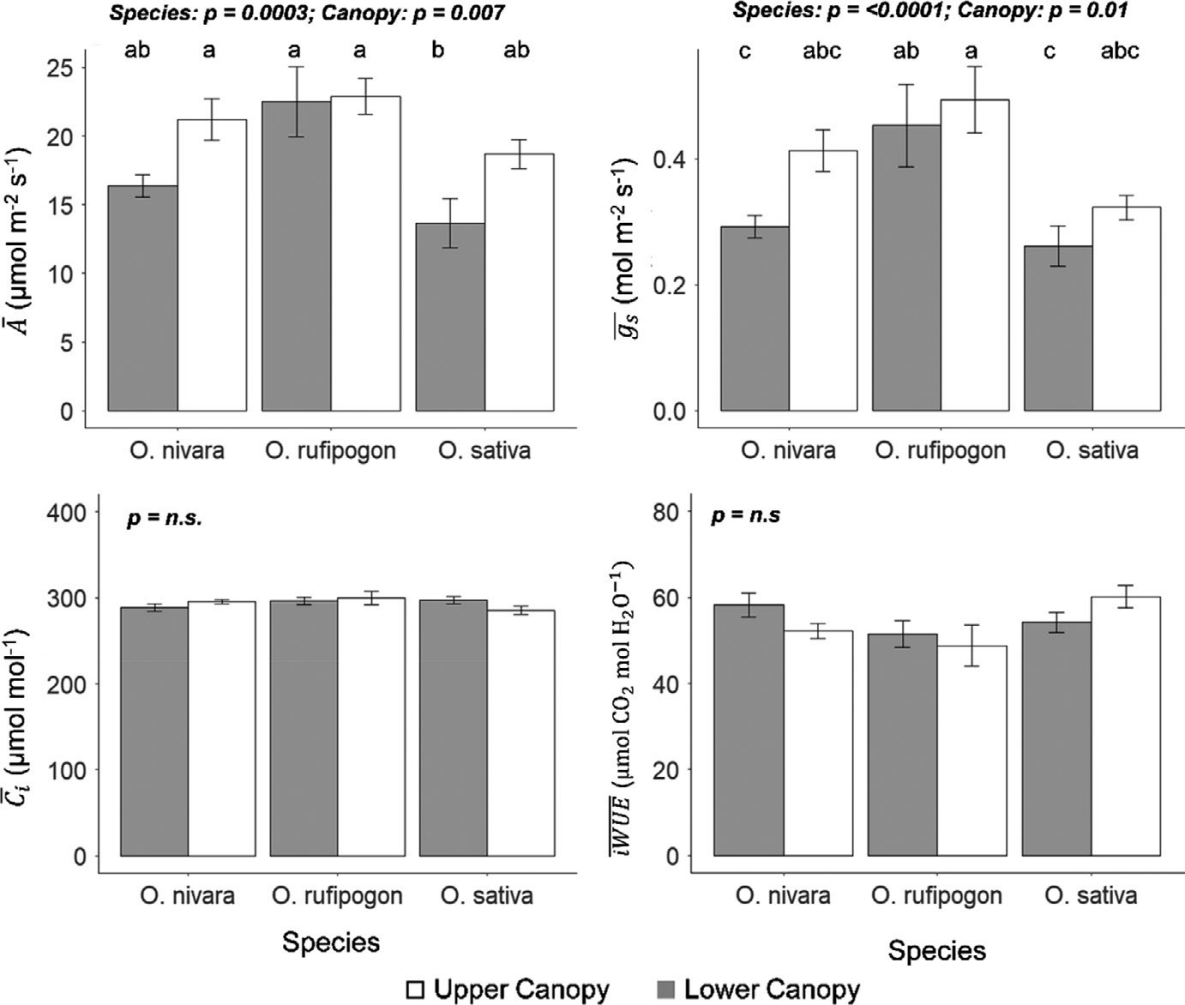
Mean performance and standard error for average CO_2_ uptake ($\bar{A}$), average stomatal conductance (${\bar{g}}_{s}$), average intercellular CO_2_ concentration (${\bar{C}}_{i}$), and average intrinsic water‐use efficiency ($\bar{\mathrm{iWUE}}=\bar{A}/{\bar{g}}_{s}$) during photosynthetic induction. These traits were measured in three *Oryza* species (*O*. *sativa*, *O*. *rufipogon*, and *O*. *nivara*) at two canopy levels (upper and lower). Each bar is the mean of six plants (*n* = 6) ± SE.

**FIGURE 3:**
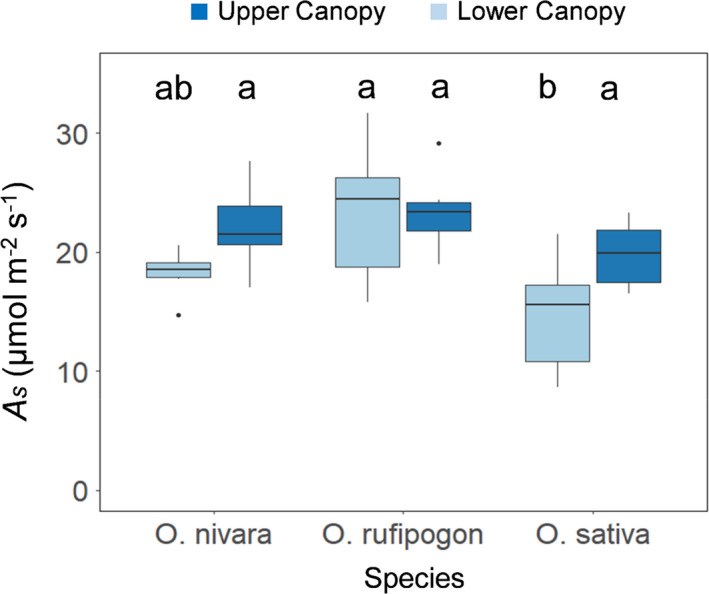
CO_2_ uptake once it has reached a steady‐state rate after photosynthetic induction (A_s_) in three *Oryza* species (*O*. *sativa*, *O*. *rufipogon*, and *O*. *nivara*) and two canopy levels (upper and lower). Letters are indicative of significant differences between treatments. Six plants were measured per boxplot (*n* = 6).

Significant differences were found among accessions for average CO_2_ uptake ($\bar{A}$) and stomatal conductance during induction (${\bar{g}}_{s}$) (Figures 1, 2, Table 2). Although the rates in the upper canopy leaves after 20 min of high light are very similar across the three accessions, the time taken to reach this point differs greatly, with the *O*. *sativa* accession clearly being the slowest (Figure 1). The *O*. *rufipogon* accession was the top‐performing accession for $\bar{A}$ (accession mean: 22.7 µmol m^−2^ s^−1^), also achieving the highest steady‐state *A* after the induction period (*A_s_*) (accession mean: 23.4 µmol m^−2^ s^−1^) (Figures 1, 2, 3). The lowest‐performing accession for *A* during induction was *O*. *sativa* with an average of 16.2 µmol m^−2^ s^−1^ for $\bar{A}$ and 17.3 µmol m^−2^ s^−1^ for *A_s_*, respectively, 40% and 35% lower than the *O*. *rufipogon* accession (Figures 1, 2, 3). Additionally, leaf position in the canopy had a significant effect on $\bar{A}$ and ${\bar{g}}_{s}$, with these traits being, respectively, 19% and 22% higher in the upper canopy level during the transition from low to high light (Figure 2). Overall, the lowest‐performing leaves were those of the lower canopy of the *O*. *sativa* accession with a mean $\bar{A}$ of 13.6 µmol m^−2^ s^−1^ (Figure 2). *O*. *sativa* also had the lowest ${\bar{g}}_{s}$ (Figure 2).

**TABLE 2:** Summary statistics and analysis of variance (ANOVA) for measured photosynthetic and physiological parameters

Light Environment	Trait	Min	Max	Average	SE (±)	Species (*p*‐val)	Canopy (*p*‐val)	Species ✕ Canopy (*p*‐val)
Steady‐state	*A*_sat_	14.2	30.8	23.5	0.7	**0.04**	0.88	0.45
	*g_s_*	0.188	1.727	0.714	0.06	**0.009**	0.13	0.14
	*C_i_*	199.6	338.7	296.2	5.7	**0.01**	0.08	0.35
	iWUE	14.27	127.36	43.05	4.2	**0.007**	0.51	0.37
	CE	0.07	0.28	0.13	0.007	**0.0001**	0.68	0.64
	*Γ*	45.9	83.55	55.88	1.2	0.77	0.14	0.46
	*A*_max_	18.7	46.9	33.5	1.2	**0.005**	0.57	0.87
	*V_c_*_,__max_	69	220	130	5.9	**0.006**	0.19	0.75
	*J*_max_	88	267	165.1	7	**0.001**	0.13	0.67
	TPU	6.7	17.4	11.7	.4	**0.0006**	0.21	0.61
	*L*	0.06	0.42	0.18	0.02	0.16	0.47	0.54
Non‐steady‐state	$\bar{A}$	7.8	31.1	19.2	0.8	**0.0003**	**0.007**	0.21
	$\overset{‐}{g_{s}}$	0.13	0.71	0.37	0.02	**<0.0001**	**0.01**	0.54
	$\overset{‐}{C_{i}}$	271	325.5	293.7	2	0.27	0.87	0.09
	$\bar{\mathrm{iWUE}}$	32.9	69.4	54.1	1.3	0.07	0.72	0.14
	*A_s_*	8.64	31.65	20.27	0.8	**0.001**	**0.01**	0.23
	*IT_50_* *_A_*	0.04	5.47	1.19	0.17	0.33	0.11	0.46
	*IT_90_* *_A_*	1.21	11.37	4.68	0.44	**0.01**	0.07	0.66
	$g_{s\;50\;i}$	0.12	5.24	1.64	0.24	0.56	0.58	0.33
	$g_{s\;90\;i}$	0.15	10.55	4.28	0.49	**0.03**	0.08	**0.04**
	$g_{s\;50\;r}$	2.36	7.58	4.8	0.26	0.009	0.32	.35
	$g_{s\;90\;r}$	4.79	12.97	8.88	0.37	0.002	0.49	.74
	NPQ	1.54	2.5	2.05	0.04	**0.04**	0.88	0.46
Leaf Traits	Leaf Area	25.99	52.66	40.29	1.26	**<0.0001**	0.08	0.2
	Dry Leaf Weight	0.12	0.32	0.2	0.009	**0.002**	**0.02**	0.17
	SLA	148.3	335.4	205.5	7.13	**<0.0001**	0.11	0.41

Min, minimum value; max, maximum value; SE, standard error.

Bold values indicate statistical significant value (*p* < 0.05).

**FIGURE 4:**
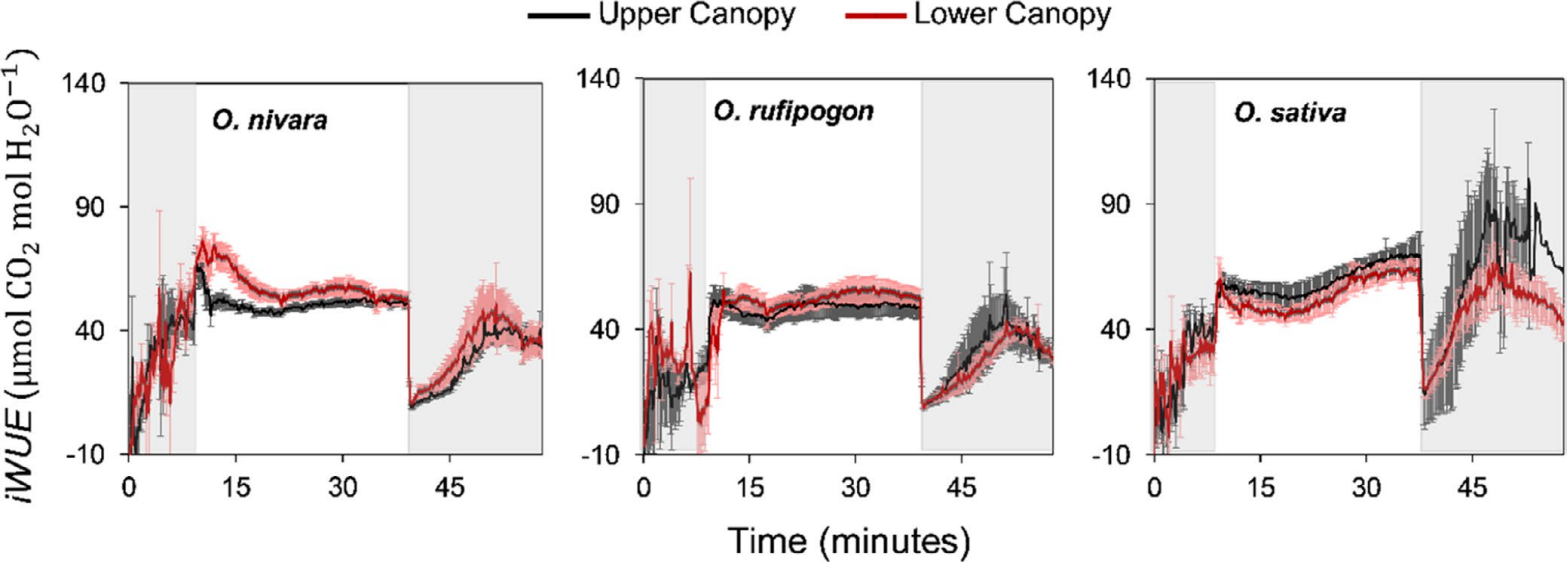
Intrinsic water‐use efficiency (iWUE = A/*g_s_*) over time during photosynthetic induction (the transition from low light (50 µmol m^−2^ s^−1^) to high light (1,500 µmol m^−2^ s^−1^) and photosynthetic relaxation (the transition from high light to low light). Periods of low light are shown by the gray areas in the figure, and highlight is shown in white. This measurement was taken at an ambient [CO_2_] of 400 µmol mol^−1^ on three *Oryza* species and at two canopy levels. Each point is the mean (± SE) of six plants (*n* = 6)

However, no significant differences were found for average intercellular CO_2_ concentration (${\bar{C}}_{i}$) (Figure 2, Figure S2). Consistent with this, there were no significant differences (*p* > 0.05) between accession or canopy levels for intrinsic water‐use efficiency (*iWUE* = A/*g_s_*) during induction (Figures 2, 4). However, the *O*. *sativa* accession had *iWUE* twice as high relative to the other two accessions during the transition from high light to low light caused by lower levels of stomatal conductance (Figure 4).

**FIGURE 5:**
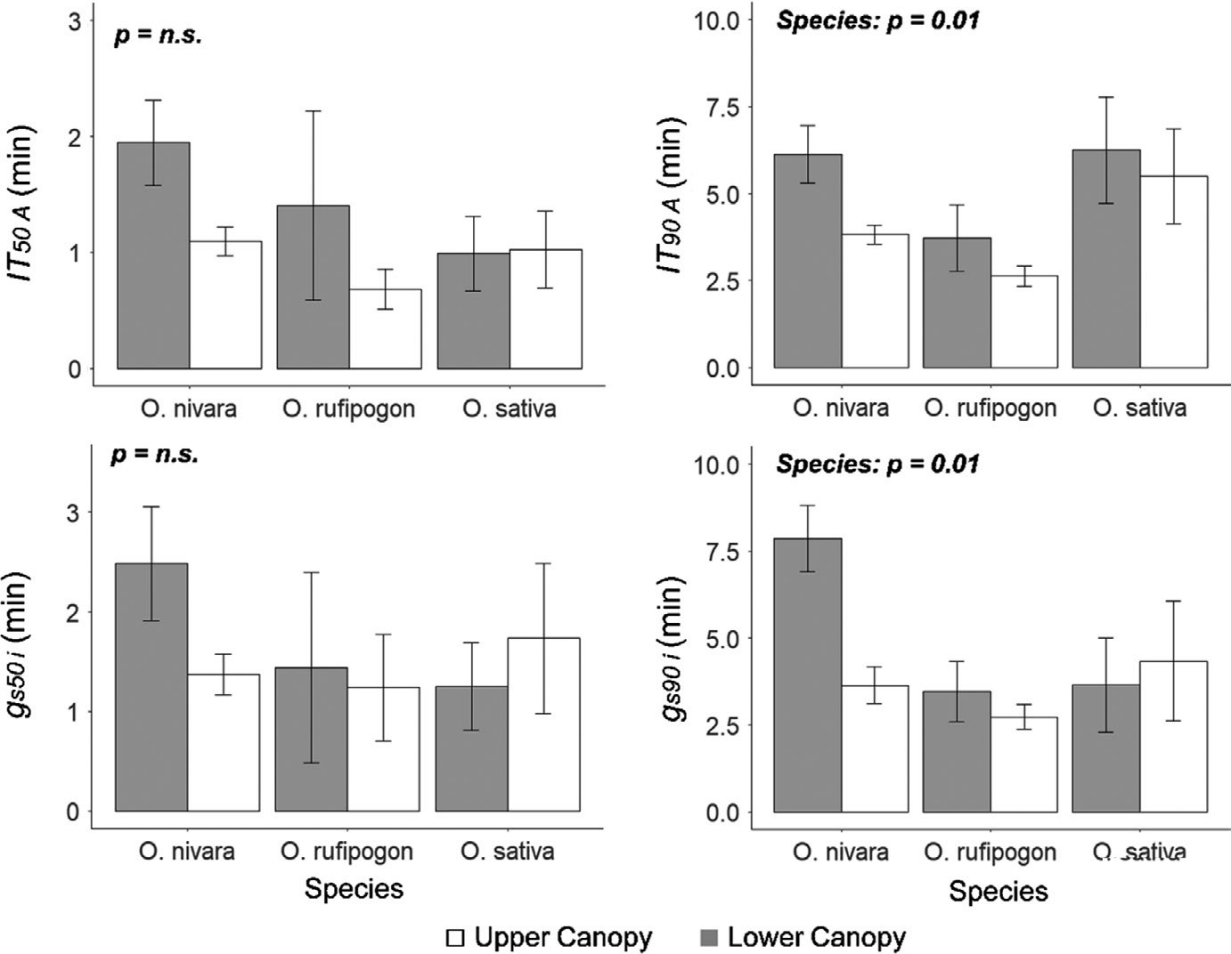
Mean performance and standard error for time to 50% induction of CO_2_ uptake (*IT_50_* *_A_*), time to 90% induction of CO_2_ uptake (*IT_90_* *_A_*), time to 50% induction of stomatal conductance (*g_s50_* *_i_*), and time to 50% induction of stomatal conductance (*g_s90_* *_i_*) during photosynthetic induction. These traits were measured in three *Oryza* species (*O*. *sativa*, *O*. *rufipogon*, and *O*. *nivara*) at two canopy levels (upper and lower). Each bar is the mean of six plants (*n* = 6) ± SE.

The speed of induction was quantified as the time to reach 50% and 90% of steady‐state *A* during induction (*IT_50_* *_A_* and *IT_90_* *_A_*, respectively). While not significant, *IT_50_* *_A_* was c.a. 1 min slower in the lower canopy leaves of the *O*. *rufipogon* and *O*. *nivara* accessions than in the *O*. *sativa* accession. However, *IT_50_* *_A_* was about equal between canopy levels of the *O*. *sativa* accession (Figure 5). When measured over the longer time period (*IT_90_* *_A_*), the *O*. *rufipogon* accession was significantly faster at 190 s compared to 350 s for the *O*. *sativa* accession (Figure 5). The speed of stomatal opening was also evaluated by measuring the time to reach 50% and 90% of steady‐state stomatal conductance (*g_s_*) (*g_s_* *_50_* *_i_* and *g_s_* *_90_* *_i_*_,_ *respectively*). As with *IT_50_* *_A_*, no significant differences were found between accession or canopy levels for *g_s_* *_50_* *_i_* (Figure 5). However, for *g_s_* *_90_* *_i_*_,_ the *O*. *nivara* accession was significantly slower than the *O*. *rufipogon* accession, mostly due to a very slow stomatal response (taking 7.5 minutes) in its lower canopy leaves (Figure 5). There were strong similarities between *IT_50_* for *A* and *g_s_* and then between *IT_90_* for *A* and *g_s_* indicating a strong coupling between the two parameters, and consistent with the lack of difference in ${\bar{C}}_{i}$ (Figure 5). The speed of induction was also examined by comparing the slope of CO_2_ uptake during the initial 5 minutes of induction (Figure S3). These results further suggest what was seen for *IT_50_* *_A_*, *IT_90_* *_A_*, *g_s_* *_50_* *_i_* and *g_s_* *_90_* *_i_*, where the *O*. *rufipogon* and *O*. *nivara* accessions had significantly faster induction responses than the *O*. *sativa* accession.

#### 3.1.1 Steady‐state measurements of photosynthesis and its limitations

**FIGURE 6:**
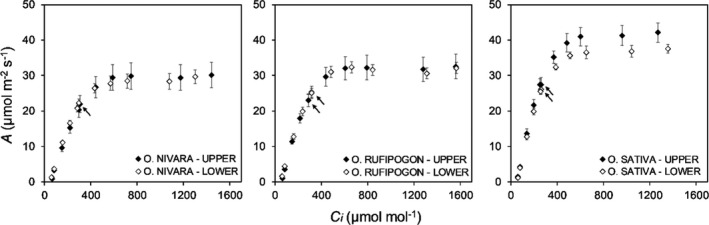
Response of CO_2_ uptake (*A*) to intercellular [CO_2_] (*C_i_*) in three *Oryza* species (*O*. *sativa*, *O*. *rufipogon*, and *O*. *nivara*) measured at two canopy levels (upper and lower). The CO_2_ response curves were measured in saturating light conditions (1,500 µmol m^−2^ s^−1^). Each point is the mean (±SE) of six plants (*n* = 6).

**FIGURE 7:**
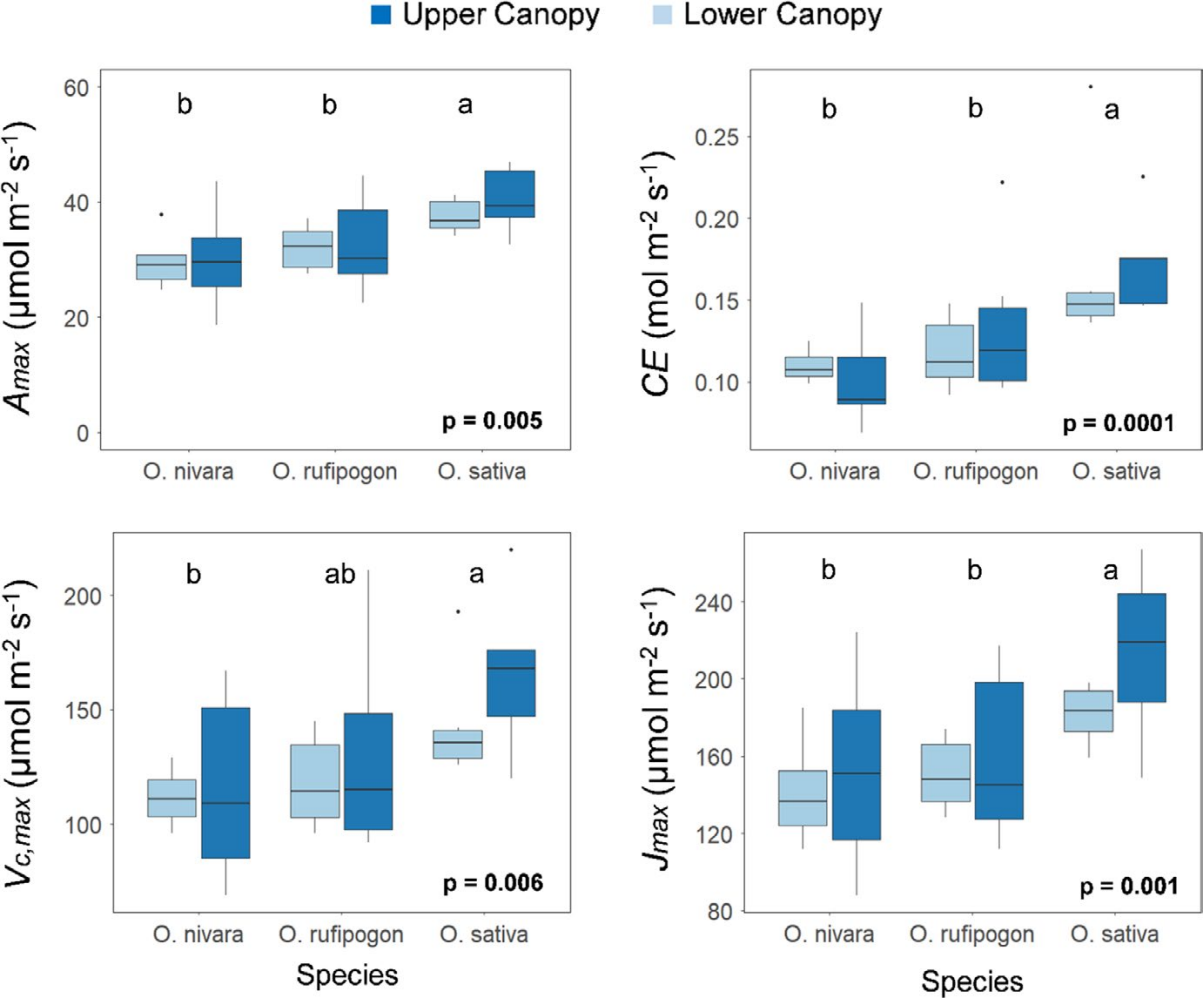
Variation for CO_2_ uptake in saturating light and [CO_2_] (*A*_max_), carboxylation efficiency (*CE*), the maximum rate of carboxylation efficiency (*V_c_*_,__max_), and the maximum rate of electron transport (*J*_max_) in three *Oryza* species and two canopy levels. Letters are indicative of significant differences between species. Each boxplot represents six plants (*n* = 6)

The responses of *A* to *C_i_* (*A*/*C_i_*) were measured to allow the analysis of limitations in the three accessions. There were significant differences across accessions in parameters derived from the *A*/*C*_i_ responses: carboxylation efficiency (*CE*), the maximum rate of CO_2_ uptake in saturating light and [CO_2_] (*A*_max_), the maximum rate of carboxylation (*V_c_*_,__max_), the maximum rate of electron transport (*J*_max_), and triosephosphate utilization limitation (TPU) (Figures 6, 7, Table 2). *O*. *sativa* had significantly higher *CE* (accession mean of 0.17 mol m^–2 ^s^–1^) relative to the other two accessions (accession means of 0.10–0.12 mol m^–2 ^s^–1^) (Figure 7). The *O*. *sativa* accession also had significantly higher values than either the *O*. *rufipogon* or *O*. *nivara* accessions for *A*_max_, *V_c_*_,__max_, *J*_max_, and TPU, indicating higher photosynthetic capacity in all aspects of photosynthetic carbon metabolism and electron transport (Figure 7). No significant differences were found across accession for compensation point (*Γ*) or stomatal limitation (*L*) (Table 2). Additionally, there were no significant differences between the two canopy levels for any of these traits (Table 2). Through the analysis of the *A*/*C_i_* curves, it was found that the three accessions were predominately limited by Rubisco rather than the regeneration of RuBP (Figure 6).

#### 3.1.2 Limitations to photosynthesis during induction

**FIGURE 8:**
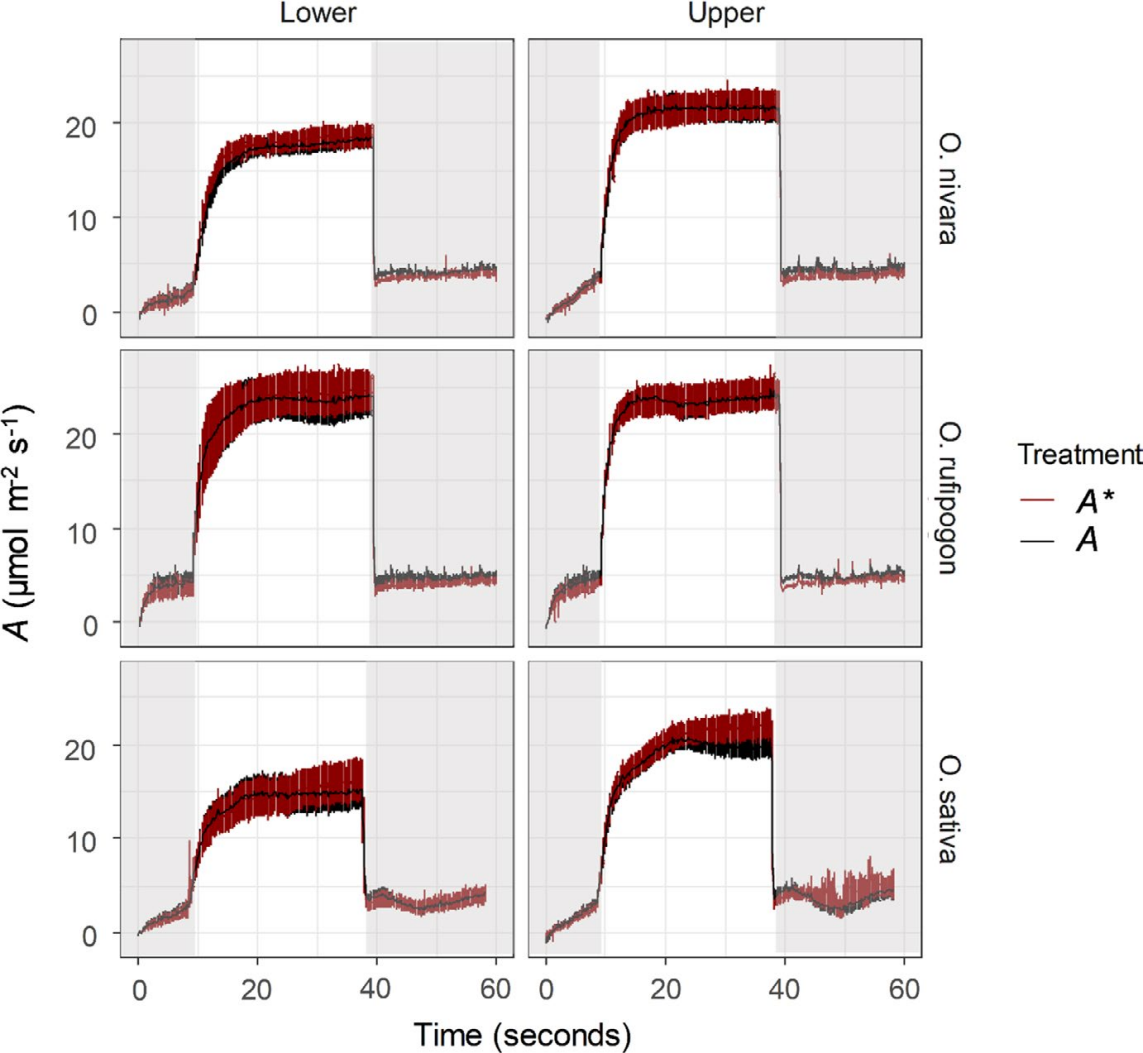
The response of uncorrected leaf CO_2_ uptake (*A*; ●) and the response of leaf CO_2_ uptake corrected for stomatal limitation (A^*^; 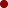) over time in different rice species and canopy levels. The vertical line indicates the mean time for the activation of Rubisco (τ) per accession. Each point represents the mean of 6 plants ± SE (*n* = 6)

CO_2_ uptake (*A*) and CO_2_ uptake corrected for stomatal limitation (A^*^) were compared over the course of photosynthetic induction and relaxation. Here, *A* and A^*^ were very similar throughout the measurement suggesting that photosynthesis was not strongly limited by stomata, but rather biochemistry (Figure 8). Only in the *O*.* sativa*, accession was there a substantial difference between *A* and A^*^, but only after *A* had reached a steady state (Figure 8). Additionally, average *C_i_* during steady‐state measurements and induction were very similar (296.2 µmol mol^−1^ and 293.7 µmol mol^−1^, respectively) (Table 2). However, *A* during induction was on average lower than at steady state (Table 2). This is also indicative of greater limitation to photosynthesis by biochemistry than stomata during induction.

#### 3.1.3 Shade to sun transition and the relaxation of NPQ

**FIGURE 9:**
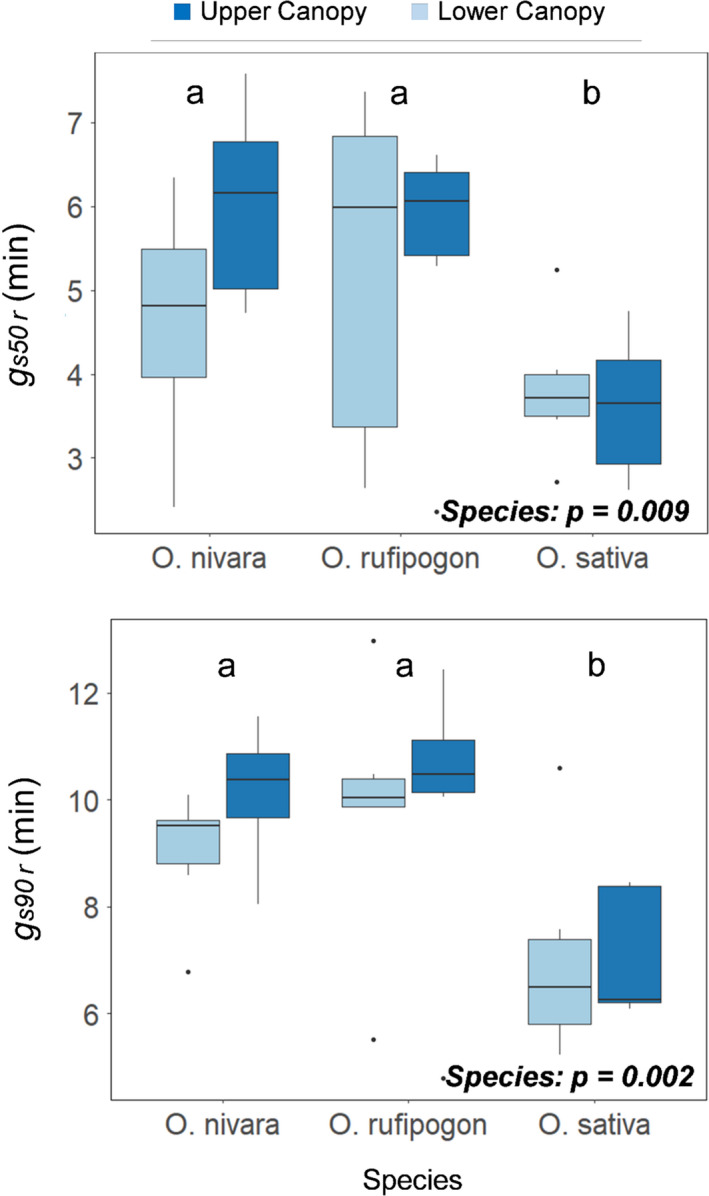
A. The time to 50% stomatal closure during photosynthetic relaxation (*g_s50_* *_r_*) in three *Oryza* species and at two canopy levels. B. The time to 90% stomatal closure during photosynthetic relaxation (*g_s90_* *_r_*) in three rice species and at two canopy levels. Letters are indicative of a significant difference between species. Six plants were measured per boxplot (*n* = 6)

During the transition from low light to high light, that is, induction, CO_2_ uptake (*A*) and stomatal conductance (*g_s_*) were strongly coupled regardless of species or canopy level (Figure 1). Following the high‐to‐low light transition, dynamics of *A* and *g_s_* were not coupled, with *g_s_* decreasing far more slowly (Figure 1). Indeed, the transition from sun to shade was characterized by a lag in response by stomata that was several minutes longer than *A* (Figure 1). The time to 50% and 90% *g_s_* during relaxation (*g_s_* *_50_* *_r_* and *g_s_* *_90_* *_r_*, respectively) was calculated to evaluate the speed of stomatal closure in response to the change from high light to low light. *g_s_* *_50_* *_r_* varied from 2.36 minutes (141 s) to 7.58 minutes (454 s) across accessions and canopy levels, while *g_s_* *_50_* *_r_* varied from 4.79 minutes to 12.97 minutes (Table 2). In both *g_s_* *_50_* *_r_* and *g_s_* *_50_* *_r_*_,_ the *O*. *sativa* accession was significantly faster than both wild rice accessions, taking c.a. 200 s and 240–260 s less to reach *g_s_* *_50_* *_r_* and *g_s_* *_50_* *_r_*, respectively (Figure 9).

**FIGURE 10:**
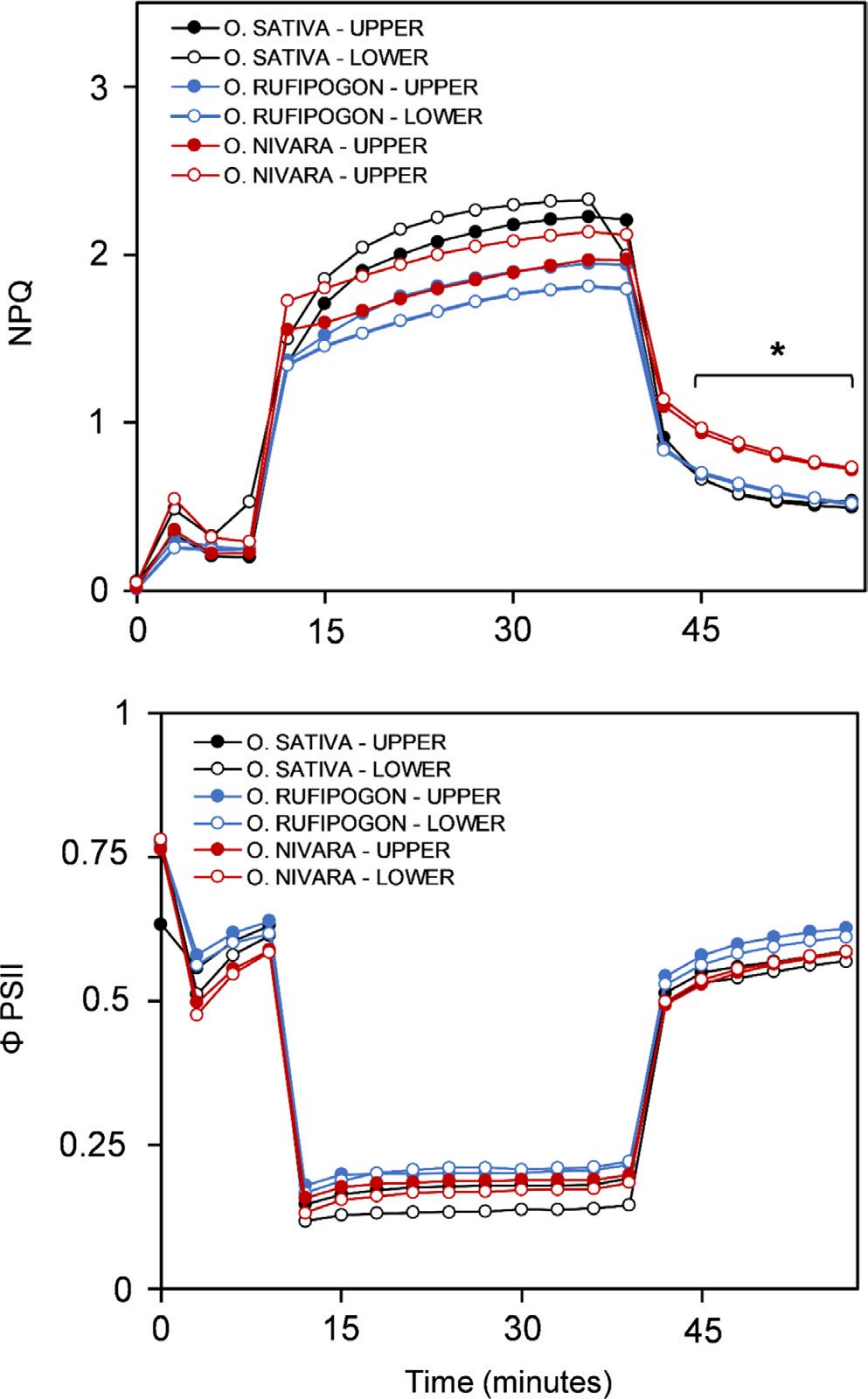
Non‐photochemical quenching (NPQ) during photosynthetic induction and relaxation measured in three *Oryza* species at two canopy levels. Each point is the mean of 6 plants (*n* = 6); *p* < 0.05.

In addition to the speed of the response of stomata, NPQ was also calculated from chlorophyll fluorescence data collected throughout the entire measurement. The *O*. *sativa* accession also had significantly higher levels of NPQ than the *O*. *rufipogon* and *O*. *nivara* accessions (Figure S4). Additionally, significant differences between species were seen for NPQ relaxation, with the *O*. *nivara* accession relaxing the slowest out of the three accessions (Figure 10).

#### 3.1.4 Differences among accessions for leaf traits

Significant differences were also found for leaf area (LA), dry leaf weight (DW), and specific leaf area (SLA), which is a ratio of LA to DW (Table 2, Figure S5). All three traits were significantly different between accessions, while only DW was significantly different as well between canopy levels (Table 2). *O*. *nivara* had significantly lower LA than both *O*. *rufipogon* and *O*. *sativa* (Figure S5). However, *O*. *rufipogon* had significantly higher *DW* than the two other accessions (Figure S5). Finally, *O*. *sativa* had the highest values for SLA (Figure S5).

## 4 DISCUSSION

This project aimed to characterize both the steady‐ and non‐steady‐state photosynthetic responses within three species of the *Oryza* genus at different levels within the plant canopy. Here, significant variation was found for photosynthetic traits across the species, such as photosynthetic traits related to induction, stomatal opening and closing, NPQ, photosynthetic capacity, and leaf traits. Generally, the wild rice accessions showed faster induction‐related traits. The cultivated rice accession, however, had significantly faster stomatal closure during transitions from high light to low light, resulting in increased *iWUE* relative to the wild rice accessions. Furthermore, differences were found for non‐steady‐state photosynthetic traits between different canopy levels, with the upper canopy significantly outperforming the lower canopy for traits such as $\bar{A}$, *A_s_*, and *IT_90_* *_A_*. These results could have implications for how we seek to model non‐steady‐state photosynthesis within different levels of the rice canopy. Additionally, this work suggests that wild rice species could be helpful in improving cultivated rice photosynthetic induction, which could translate into increased rice productivity in the future.

### 4.1 Wild rice accessions perform better in photosynthetic induction‐related traits relative to the *O. sativa* accession, and significant variation exists between accessions for photosynthesis related traits

Both the *O*. *rufipogon* and *O*. *nivara* accessions had higher rates for *A* than the *O*. *sativa* accession during non‐steady‐state photosynthesis measurement (Figures 1, 2) likely due to their faster induction response (Figure S3). Wild rice species are often utilized as sources of novel germplasm to improve cultivated rice for increased tolerance to biotic and abiotic stresses. These two wild accessions, therefore, suggest a resource for breeding increased speed of photosynthetic induction into cultivated rice.

Here, only single accessions are compared with IR64, which has been the basis for breeding many of the most used current *indica* rice lines (Mackill & Khush, 2018). Even faster rates of induction might be found by exploring more accessions of these two wild relatives. The finding raises the question: are some wild rice species more adept at coping with fluctuating light environments? Here, the *O*. *rufipogon* accession had the fastest *IT_50_* *_A_*, *IT_90_* *_A_*, *g_s50_* *_i_*, and *g_s90_* *_i_* response out of the measured accessions (Figure 5). This supports recent evidence where *O*. *rufipogon* and other wild rice species had a faster *A* and *g_s_* response during photosynthetic induction when compared with domesticated rice (Zhang et al., 2019). Additionally, it was previously shown that *O*. *rufipogon* has a higher Rubisco activase (Rca) content and Rubisco activation state relative to *O*. *sativa* (Masumoto et al., 2005). Rubisco activase plays a central role in non‐steady‐state photosynthesis, including photosynthetic induction (Yamori et al., 2012). This may partially explain why *O*. *rufipogon* induces more rapidly than *O*. *sativa* accession under non‐steady‐state conditions, since the speed of induction, in the absence of stomatal limitation as found here, is associated with the amount of Rca (Carmo‐Silva et al., 2013). Meanwhile, a higher Rubisco activation state will allow a more rapid initial increase in the CO_2_ uptake rate during induction, as seen in Figure 1 (Carmo‐Silva et al., 2013).

This ability to respond to quick changes in irradiance may be particularly beneficial for wild rice species that grow in diverse natural ecosystems where there is competition for light not just within their own canopy but also from surrounding vegetation (Morishima et al., 1984; Yamanaka et al., 2003). Inadvertent selection may have been made during the domestication process in favor of plants that are less responsive to changes in light but have an overall higher photosynthetic capacity, which could then contribute to higher yields. Previously, it has been hypothesized that there is a tradeoff between the amount of Rubisco and Rca in the leaf (Mott & Woodrow, 2000; Woodrow & Mott, 1989). Both represent a significant amount of leaf total protein, and more Rca is commonly associated with less Rubisco, and vice versa (Fukayama et al., 2012; Jin et al., 2006).

All three species achieved similar light‐saturated rates of leaf CO_2_ uptake (Figures 1, 2), and Figure 6 shows that in each case, *in vivo* Rubisco activity was the limiting factor. This is consistent with the recent finding that rice overexpressing Rubisco resulted in increased photosynthetic capacity and yields (Yoon et al., 2020). The mass of leaf per unit area was significantly and 40% lower in *O*. *sativa* (Figure 5). Therefore, if expressed on a mass basis, *O*. *sativa* light‐saturated photosynthesis is 40% higher than the two wild accessions. This suggests that the development of the cultivated form has resulted in thinner leaves, yet without loss of steady‐state photosynthetic capacity per unit leaf area. Thus, Rubisco content has been maintained despite the reduction in leaf mass, but likely at the cost of other proteins, which may include Rca (Fukayama et al., 2012; Jin et al., 2006). However, achieving more area per unit leaf mass would have allowed more leaf area and light capture, which could outweigh the loss of efficiency per unit leaf area.

The limitations to photosynthetic induction seem to be species dependent. For example, it was shown that photosynthetic induction in soybeans is primarily limited by biochemistry, while induction in cassava is heavily limited by stomata. In rice, it might not be as clearly defined (De Souza et al., 2020; Soleh et al., 2016). Previously, it was shown that photosynthetic induction in rice is heavily limited by stomata (Yamori et al., 2020) as well as biochemistry (Acevedo‐Siaca, Coe, Quick, et al., 2020; Acevedo‐Siaca, Coe, Wang, et al., 2020). Indeed, previous work has shown that QTLs that increase stomatal conductance in rice can increase the initial slope of CO_2_ assimilation during induction (Adachi et al., 2019). This is similar to what was shown recently in *Arabidopsis* where stomatal “stay‐open” genes reduced stomatal limitation and consequently increased the rate of CO_2_ assimilation during induction (Kimura et al., 2020). However, it is possible that limitations to induction in rice may be accession or subpopulation dependent. For example, there is evidence that stomatal opening and closing can vary by rice subpopulation (Qu et al., 2016), with japonica accessions having an overall slower stomatal response. Additionally, none of the accessions utilized in these independent photosynthetic induction studies have overlapped (Acevedo‐Siaca et al., 2020b; Acevedo‐Siaca, Coe, Wang, et al., 2020; Adachi et al., 2019; Yamori et al., 2020). Furthermore, the release date for the accessions utilized in different induction studies in rice vary greatly (Acevedo‐Siaca et al., 2020a, 2020b; Yamori et al., 2020). Currently, it has not been examined how cultivar release date affects photosynthetic induction response. It is possible that release date could affect photosynthetic performance, as was seen previously in wheat under steady‐state conditions (Driever et al., 2014). In this quickly developing field of understanding photosynthesis under fluctuating light, there is still much opportunity to understand the nuances of this process and how it may differ between accessions.

### 4.2 *O*. *sativa* accession has a faster stomatal closure response during high‐to‐low light transitions relative to wild rice accessions

The coordination between *A* and *g_s_* can decrease in response to changes in light intensity, resulting in greater water loss through transpiration (McAusland et al., 2016; Qu et al., 2016; McAusland et al., 2020). In rice, *A* and *g_s_* are strongly coupled during induction (McAusland et al., 2016; Zhang et al., 2019), but can become less coordinated during the transition from high‐to‐low light (McAusland et al., 2016). This study supports previous work as *A* responded immediately to the change in light, while stomatal closure to reach the low‐light steady‐state *g_s_* took several minutes (Figures 1, 9).

The *O*. *sativa* accession had a significantly faster stomatal closure response than the *O*. *rufipogon* or *O*. *nivara* accessions (Figure 9), which resulted in higher *iWUE* and greater water conservation when the plant re‐entered low‐light conditions (Figure 4)—similar to what has been shown previously in wheat (McAusland et al., 2020). Accessions with a slower stomatal closure response saw tradeoffs with intrinsic water‐use efficiency (Figure 4), which can result in lower drought resistance (Qu et al., 2016; McAusland et al., 2020). However, it was previously shown that a slower stomatal response may be beneficial to shade‐adapted plants as it allows them to respond faster to subsequent light flecks and assimilate CO_2_ more quickly (Deans, Brodribb, et al., 2019). Consequently, it is possible that the slower stomatal response seen in the wild rice accessions is an adaptation that can help the plant cope with a more limited light environment due to increased competition for light and nutrient resources with other plants (Mohapatra et al., 2011; Wang, Burgess, et al., 2020; Waters et al., 2012). This is in strong contrast to the agricultural field setting where domesticated rice is grown with little light competition from other plant species. However, since only one accession of each wild species was examined, this does not remove the possibility that other wild accessions might be found that are superior to *O*. *sativa* in this trait.

Photosynthetic induction and relaxation will occur many times a day due to fluctuations in light within the crop canopy (Zhu et al., 2000, 2004). Each one of these changes in light presents an opportunity to improve CO_2_ uptake and water‐use efficiency, both of which can improve overall crop productivity and sustainability. As drought occurrence is expected to increase in the future due to climate change (Li et al., 2015; Wassmann et al., 2009), it is important to take into consideration avoidable water loss through transpiration to minimize irrigation requirements. Rice is a tremendously water‐intensive crop due to its cultivation in irrigated rice paddies (Bouman & Toung, 2001; Dawe et al., 2005; Wu et al., 2017). Since most breeding will have been undertaken in paddy conditions and without selection for water conservation, it is then surprising that the cultivated form was nevertheless the most water‐use efficient under these fluctuating light conditions.

### 4.3 *O*. *sativa* accession has greater photosynthetic capacity than wild relative accessions, faster NPQ relaxation

Despite the wild rice accessions performing better under non‐steady‐state conditions, the *O*. *sativa* accession had significantly higher maximum photosynthetic capacity at CO_2_ and light saturation (*A*_max_), carboxylation efficiency, NPQ, and NPQ relaxation relative to at least one wild rice accession (Figure 10, Figure S4). These results coincide with what has previously been reported in wheat, and where elite varieties have higher NPQ and faster NPQ relaxation relative to wild accessions (McAusland et al., 2020). Higher NPQ in cultivated rice could allow the plant to cope more adequately with the direct sunlight often experienced for prolonged periods of time due to monocultural production.

Additionally, the higher *A*_max_ suggests that the *O*. *sativa* accession will attain higher photosynthetic rates than the two wild accessions as global CO_2_ concentrations continue to rise. Additionally, increased sink capacity of *O*. *sativa* due to domestication and plant breeding would allow it to use the increased [CO_2_] relative to wild rice species, which may be more sink limited and unable to fully utilize the greater [CO_2_]. This is particularly important to consider as atmospheric [CO_2_] and its effect on staple food crops will need to further be taken into consideration to ensure food security in a future climate.

### 4.4 Steady‐state photosynthesis is not significantly affected by canopy level, while non‐steady‐state photosynthesis is

Here, photosynthesis was measured in the youngest, fully expanded leaves within two levels of the rice canopy. Rice and other tillering crops provide a valuable opportunity to understand photosynthesis in similarly aged leaves at different levels of the crop canopy. This is unlike other crops, such as tobacco, that has reduced photosynthetic performance at lower parts of the crop canopy due to leaf age (Clark et al., 2021). There was no significant difference between canopy levels for photosynthetic capacity or photosynthetic traits measured in steady‐state conditions (Figure 7). However, under non‐steady‐state conditions, significant differences were found between the canopy levels with average *A* and *g_s_* being lower in the lower canopy leaves (Figure 2).

Previous studies have aimed to characterize photosynthetic induction in sun and shade leaves—shade leaves typically being lower in the canopy or understory and being more dependent upon diffuse light. Shade leaves usually assimilate less CO_2_ during induction but have faster induction times (Martins et al., 2013; Urban et al., 2007). This is similar to results here, where lower‐level canopy leaves assimilated less CO_2_ relative to upper canopy leaves of the same developmental stage (Figure 2). However, lower canopy leaves did not vary significantly from upper canopy leaves in IT_50__A_ and IT_90__A_ and in some cases were slower to respond to the change from low light to high light (Figure 5). This finding may have implications for how we create dynamic photosynthesis models in rice that aim to simulate field conditions. Currently, if lower leaves are modeled in the same way as upper canopy leaves when they are exposed to an increase in irradiance, it is possible that models are overestimating the amount of overall CO_2_ assimilation by the plant. This can then lead to inaccurate projections about crop yields and limitations in the future, which could affect our ability to sustainably meet food security goals.

## 5 LIMITATIONS TO STUDY

The main limitation of the study is that only one accession of each species was examined due to limited access to germplasm. However, this study establishes the basis that natural variation exists between these three species and that the coordination between CO_2_ uptake and stomatal conductance has been conserved in wild and domesticated rice. In the future, more effort should be placed in examining additional accessions of all species surveyed. Additional information about *Oryza* induction and NPQ relaxation could allow for further identification of promising phenotypes that could be used to improve the speed of induction in *O*. *sativa* while also shedding additional light on the evolutionary history of non‐steady‐state photosynthesis in the *Oryza* genus.

## AUTHOR CONTRIBUTIONS

LGA and SPL planned the research. SPL and WPQ supervised the project. LGA conducted the experimental work and analyzed the data. JD and RL provided technical support. LGA and SPL wrote the manuscript with the input of all the other authors.

## Supporting information

Fig S1‐S5
