# Supplementary material for: Dynamics of photosynthetic induction and relaxation within the canopy of rice and two wild relatives
Source: Food Energy Secur. 2021 May 5;10(3):e286. doi: 10.1002/fes3.286 (PMC8459282; doi:10.1002/fes3.286)
Supplement: Supplementary file 1 — Fig S1‐S5 [file FES3-10-e286-s001.docx]

**SUPPLEMENTARY MATERIALS:**

**
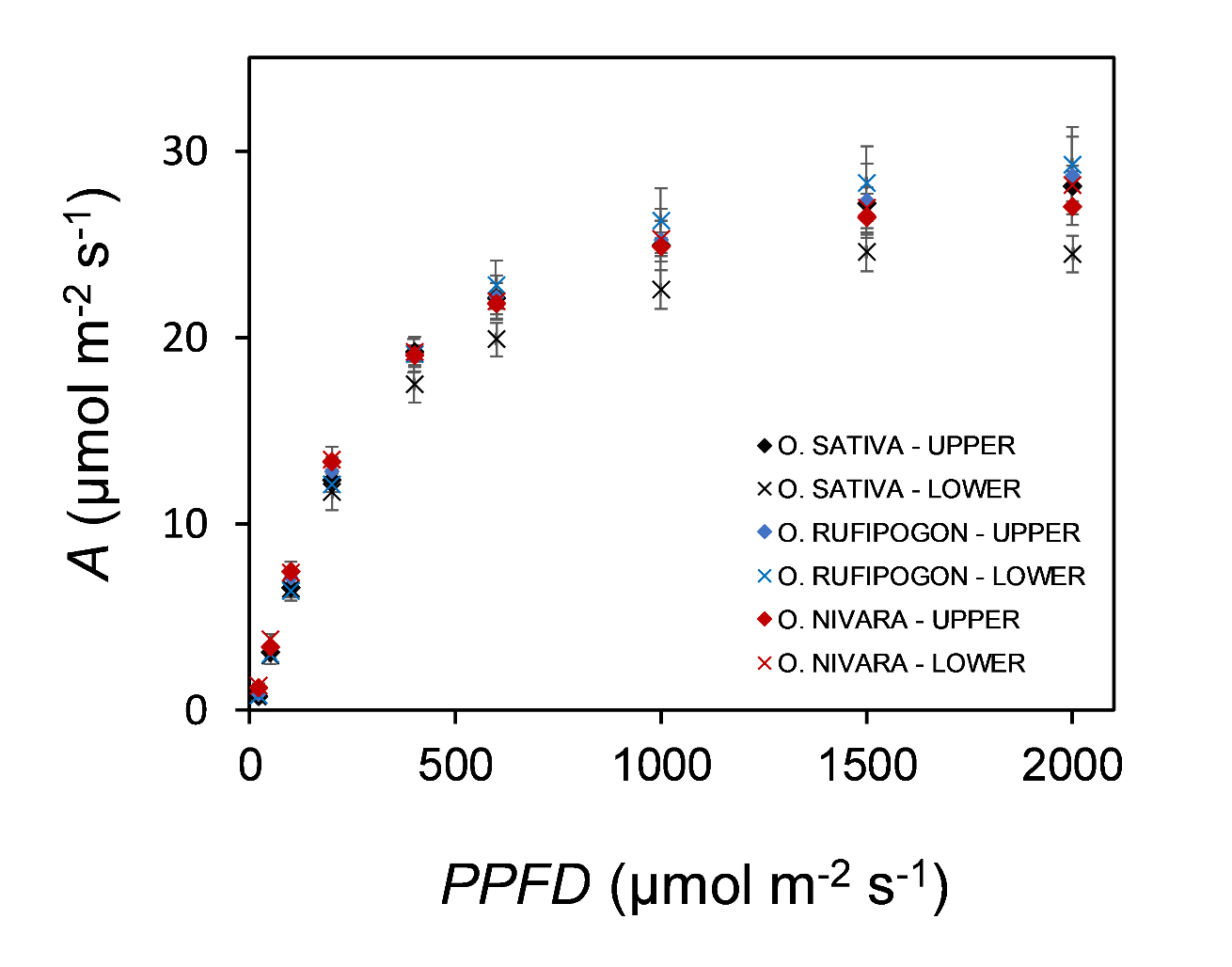
**

**Supplementary Figure 1.** The response of CO_2_ uptake (*A*) to photosynthetic photon flux density (PPFD) in three *Oryza* species (*O. sativa*, *O. rufipogon*, and *O. nivara*) measured at two canopy levels (upper and lower). The light response curves were measured in ambient CO_2_ conditions (400 µmol mol^-1^). Each point is the mean ($\pm$ SE) of six plants (n = 6).

**
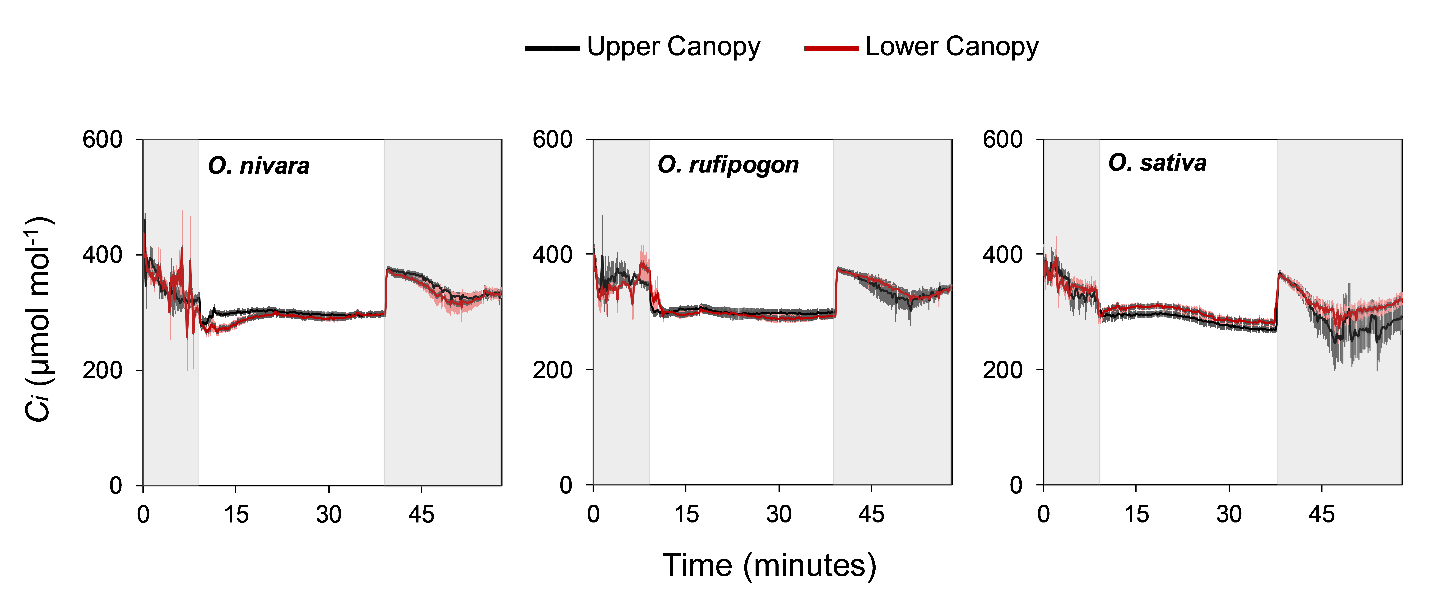
**

**Supplementary Figure 2.** Intercellular [CO_2_] (*C_i_*) over time during photosynthetic induction (the transition from low light (50 µmol m^-2^ s^-1^) to high light (1500 µmol m^-2^ s^-1^) and photosynthetic relaxation (the transition from high light to low light). Periods of low light are shown by the grey areas in the figure, highlight is shown in white. This measurement was taken at an ambient [CO_2_] of 400 µmol mol^-1^ on three *Oryza* species and at two canopy levels. Each point is the mean ($\pm$ SE) of six plants (n = 6).


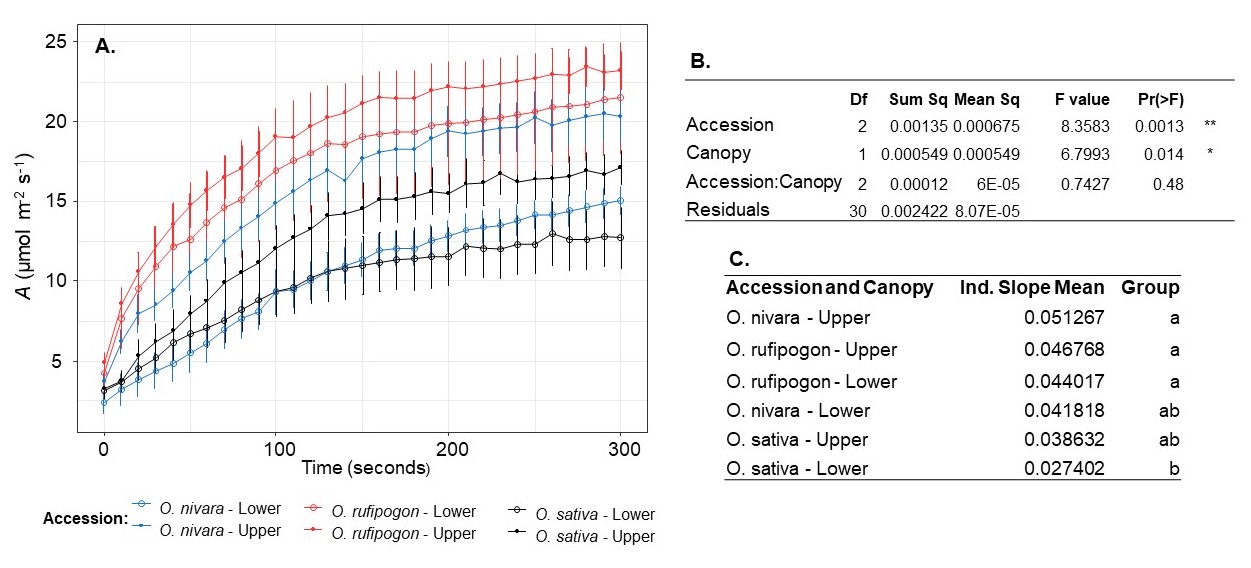


**Supplementary Figure 3.** A. The initial slope of CO2 assimilation (A) during the first five minutes of photosynthetic induction. This measurement was taken at an ambient [CO_2_] of 400 µmol mol^-1^ on three *Oryza* species and at two canopy levels. Each point is the mean ($\pm$ SE) of six plants (n = 6). B. ANOVA comparing the slope of induction response. C. Mean discrimination utilizing Tukey’s honest significant difference, where “Ind.” stands for “Induction”.


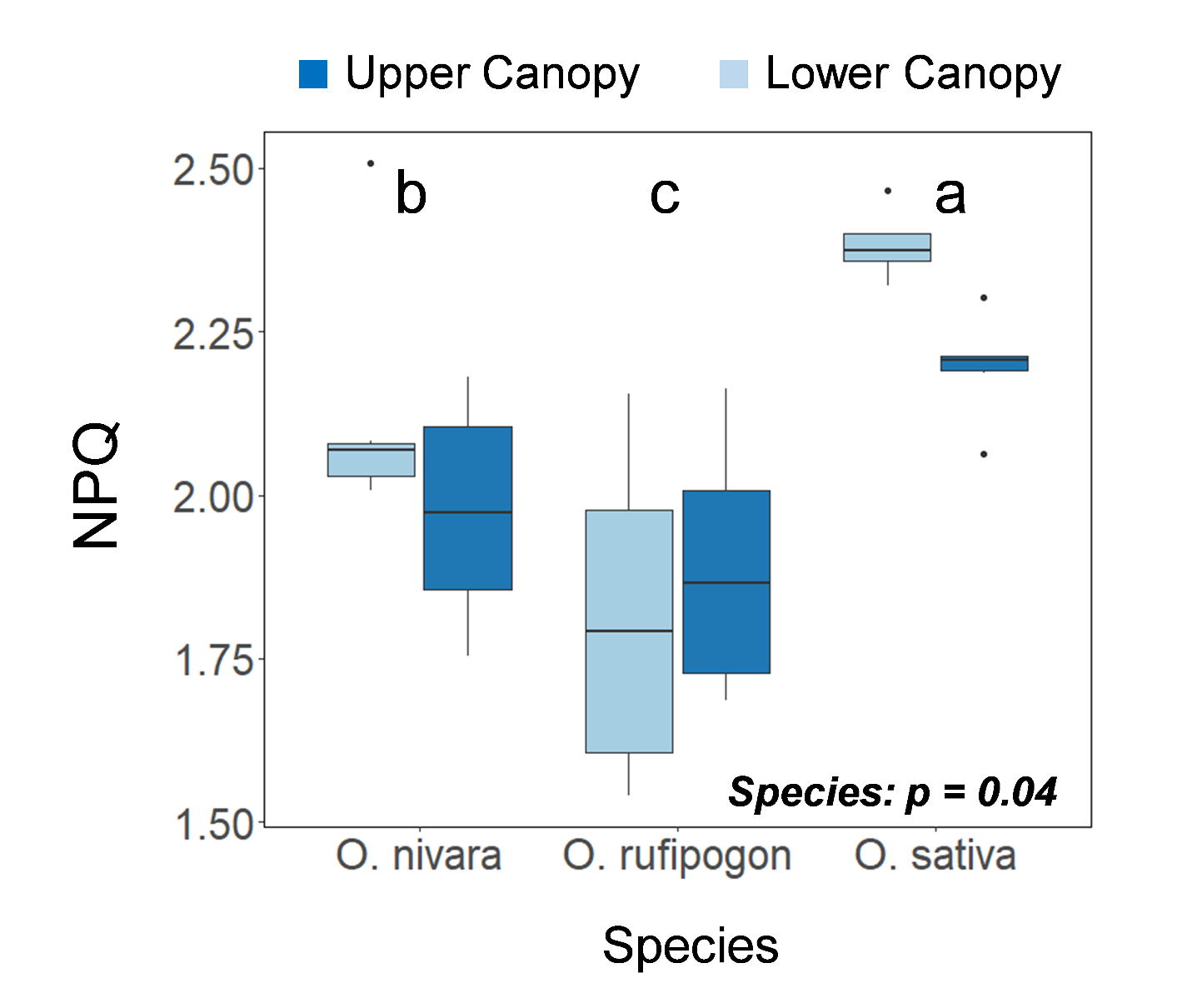


**Supplementary Figure 4.** Non-photochemical quenching (NPQ) in three rice species and at two canopy levels. Letters are indicative of a significant difference between species. Six plants were measured per boxplot (n = 6).

**
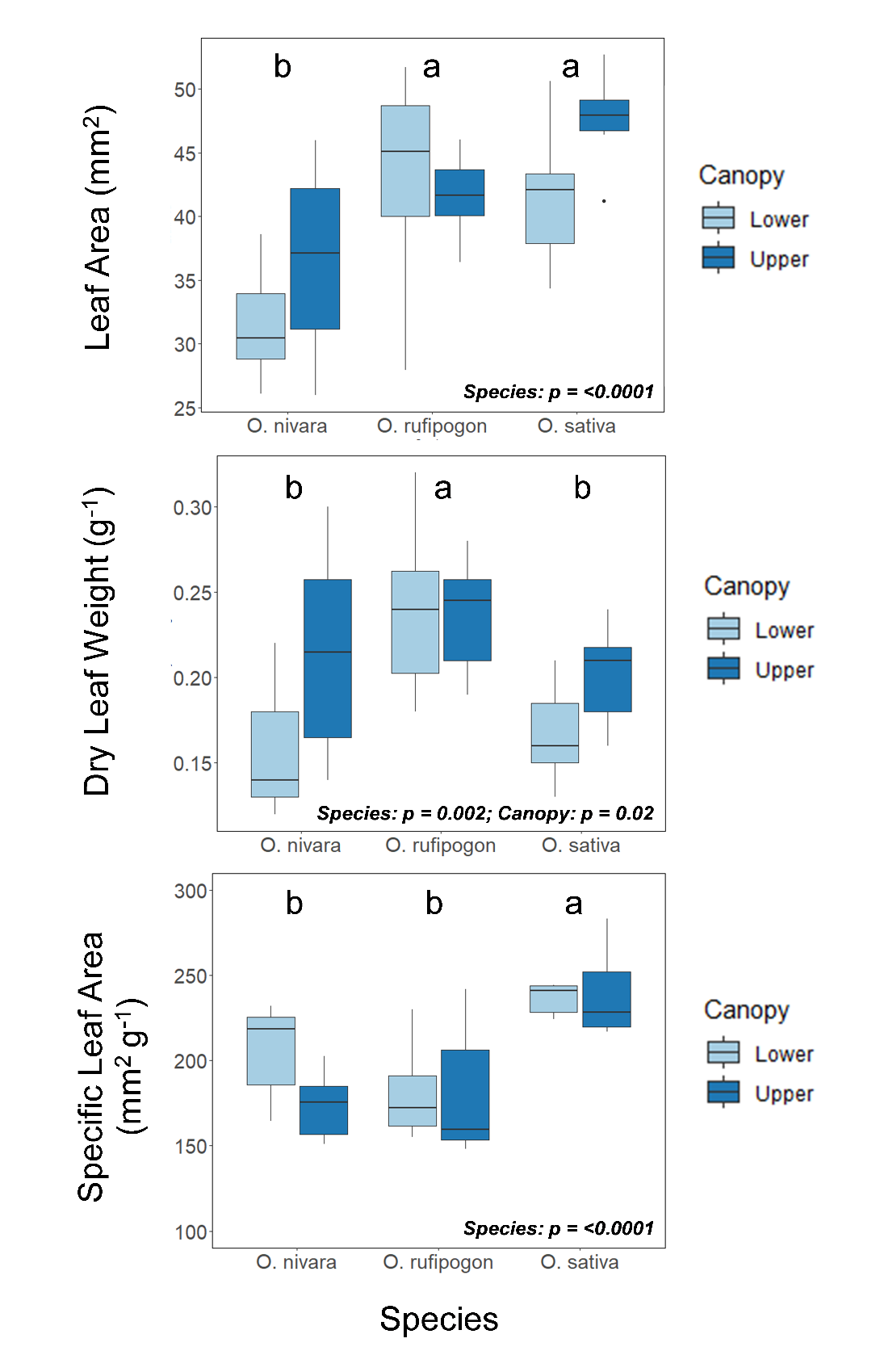
**

**Supplementary Figure 5.** Variation for leaf area (LA), dry leaf weight (DW), and specific leaf area (SLA = LA/DW) in three *Oryza* species and two canopy levels. Letters are indicative of significant differences between species. Each boxplot represents six plants (n = 6).
